# Lung Cancer Proteogenomics: Shaping the Future of Clinical Investigation

**DOI:** 10.3390/cancers16061236

**Published:** 2024-03-21

**Authors:** Theofanis Vavilis, Maria Louiza Petre, Giannis Vatsellas, Alexandra Ainatzoglou, Eleni Stamoula, Athanasios Sachinidis, Malamatenia Lamprinou, Ioannis Dardalas, Ioannis N. Vamvakaris, Ioannis Gkiozos, Konstantinos N. Syrigos, Athanasios K. Anagnostopoulos

**Affiliations:** 1 Department of Dentistry, European University Cyprus, Nicosia 2404, Cyprus; thvavilis@gmail.com; 2 Laboratory of Biology and Genetics, School of Medicine, Aristotle University of Thessaloniki, 54124 Thessaloniki, Greece; 3 Department of Biotechnology, Centre of Systems Biology, Biomedical Research Foundation of the Academy of Athens, 11527 Athens, Greece; marialouisepetre@gmail.com (M.L.P.); alexandraainatzoglou@gmail.com (A.A.);; 4 Greek Genome Centre, Biomedical Research Foundation of the Academy of Athens, 11527 Athens, Greece; gvatsellas@bioacademy.gr; 5 4th Department of Internal Medicine, Hippokration General Hospital, School of Medicine, Aristotle University of Thessaloniki, 54124 Thessaloniki, Greece; apsachini@auth.gr; 6 Department of Clinical Pharmacology, School of Medicine, Aristotle University of Thessaloniki, 54636 Thessaloniki, Greece; l.malamatenia@gmail.com (M.L.); idardalas@auth.gr (I.D.); 7 Pathology Department, “Sotiria” Hospital, 11527 Athens, Greece; i.vamvakaris@yahoo.gr; 8 Third Department of Internal Medicine, “Sotiria” Hospital, National and Kapodistrian University of Athens, 11527 Athens, Greece; yiannisgk@hotmail.com (I.G.); ksyrigos@med.uoa.gr (K.N.S.)

**Keywords:** lung cancer, NSCLC, SCLC, proteogenomics, genetic alterations, aberrant protein expression, multi-omics

## Abstract

**Simple Summary:**

Lung cancer remains the number one public health burden related to cancer worldwide. The integration of genomic profiling with in-depth proteomic profiling has introduced a new dimension of molecular cancer research, termed proteogenomics. This new-born scientific field is anticipated to fill significant knowledge gaps created by transitioning from the genome to the proteome and assist in the discovery of novel treatment pathways for lung cancer patients. This review consists of a comprehensive investigation of recent studies undertaken in the lung cancer proteogenomics setting, focusing on how elucidation of such features can evoke tangible clinical outcomes.

**Abstract:**

Background: Lung cancer is associated with a high incidence of mortality worldwide. Molecular mechanisms governing the disease have been explored by genomic studies; however, several aspects remain elusive. The integration of genomic profiling with in-depth proteomic profiling has introduced a new dimension to lung cancer research, termed proteogenomics. The aim of this review article was to investigate proteogenomic approaches in lung cancer, focusing on how elucidation of proteogenomic features can evoke tangible clinical outcomes. Methods: A strict methodological approach was adopted for study selection and key article features included molecular attributes, tumor biomarkers, and major hallmarks involved in oncogenesis. Results: As a consensus, in all studies it becomes evident that proteogenomics is anticipated to fill significant knowledge gaps and assist in the discovery of novel treatment options. Genomic profiling unravels patient driver mutations, and exploration of downstream effects uncovers great variability in transcript and protein correlation. Also, emphasis is placed on defining proteogenomic traits of tumors of major histological classes, generating a diverse portrait of predictive markers and druggable targets. Conclusions: An up-to-date synthesis of landmark lung cancer proteogenomic studies is herein provided, underpinning the importance of proteogenomics in the landscape of personalized medicine for combating lung cancer.

## 1. Introduction

Malignancies of the lungs are the second most prevalent type of cancer, in addition to being the leading cause of mortality attributed to the disease [1]. A low overall 5-year survival rate has been observed (22%), with histological type and clinical staging significantly influencing patient outcomes [2,3,4]. Two main types of lung cancer are described: non-small cell lung cancer (NSCLC) and small cell lung cancer (SCLC) [5], with the former characterized as the major subtype of neuroendocrine tumors by the 2015 WHO Classification of Lung Tumors [6]. Representing 85% of lung cancer diagnoses, NSCLC is currently further subdivided into three principal categories on the basis of histological and molecular features. Specifically, these include lung adenocarcinoma (LUAD), squamous cell carcinoma (SCC), and large cell carcinoma (LCC) [6,7].

Despite considerable regional variability, a twofold incidence of lung cancer is observed in men on a global scale [1]. Particularly in developed countries, disease epidemiology is largely driven by the tobacco epidemic [8]. Notwithstanding the significant correlation between smoking and lung cancer, it is estimated that 25% of cases occur among non-smokers [5,9,10]. Particularly in East Asia, high disease burden has been noted among non-smoking women [11,12]. Indeed, in this population group, exposure to indoor pollutants such as fumes generated by cooking oils or charcoal use for household heating have been implicated in carcinogenesis [13,14,15].

Furthermore, a striking example presented by a nationwide study conducted in Taiwan revealed that 92.1% of female lung cancer patients had never smoked. The high incidence of disease occurrence in the specific population group was partly attributed to air pollution [16]. Along with the high frequency of epidermal growth factor receptor (*EGFR*) activating mutations, the early onset of lung adenocarcinoma (LUAD) among never-smokers in this region is suggestive of a genetic contribution towards disease presentation [17]. Meta-analysis of 11 genetic loci encountered in Asian non-smoking adenocarcinoma patients discovered through genome-wide association studies (GWAS) revealed two novel SNPs (rs3817963 in *BTNL2* and rs2179920 in *HLA-DPB1*) characterized as LUAD risk factors [18]. It is, therefore, evident that distinct molecular, environmental and clinical factors underpin the etiopathology of lung cancer [19].

Within this context, the need to elucidate the molecular architecture of lung cancer in a geographically diverse setting has arisen. Identification of the tumor molecular profile/signature on an individual patient basis is crucial in the era of precision medicine [20,21]. Undoubtedly, clinical course and disease progression are defined by each patient’s unique mutational background. Principally focusing on smoking, genomic studies have provided us with extensive catalogues of somatic mutations present in lung cancer patients, linking mutational patterns to risk factors. Most notably, thus far, genomic studies have revealed the mutational background and gene expression patterns commonly found in LUAD, paving the way for advances in targeted therapeutic strategies [22,23]. While progress achieved by genomics has shed light on key molecular mechanisms governing lung cancer, the downstream effects of genetic alterations, along with drivers of drug resistance, remain poorly understood.

In an effort to address these substantial knowledge gaps regarding the biology of tumor development and drug target identification, the field of proteogenomics has emerged [24]. Encompassing the integration of next generation sequencing (NGS) technology and mass spectrometry (MS) proteomics, proteogenomics is propelling the exploration of functional impacts associated with genetic alterations, including driver mutations and chromosomal aberrations. The combined genomic profiling and proteomic and phosphoproteomic analysis of clinical samples [24,25] is anticipated to provide a holistic view of disease severity and patient management.

The number of studies aimed at elucidating the proteogenomic landscape of lung cancer, particularly LUAD, through tumor tissue analysis and by identifying its mutational profile, proteomic signatures, phosphorylation patterns and protein co-variation, is rapidly increasing.

The present review consists of a critical synthesis of landmark proteogenomic studies, providing the research community with a comprehensive map of findings on the molecular attributes of lung cancer, tumor biomarkers, and major hallmarks involved in oncogenesis and pathobiology.

## 2. Materials and Methods

The systematic review adhered to the guidelines set forth by the Preferred Reporting Items for Systematic Reviews and Meta-Analyses (PRISMA) [26]. The study was registered with OSF (Open Science Framework) Registries, DOI: (https://doi.org/10.17605/OSF.IO/3X2DH, accessed on 23 February 2024). A comprehensive search was conducted across multiple databases, including PubMed, Google Scholar, and ScienceDirect. The initial database retrieval involved screening by title and abstract, employing specific search terms such as “lung cancer”, “proteogenomics”, “multi-omics”, “LUAD”, “NSCLC”, “lung tumor”, “proteomics”, “genomics”, “phosphoproteomics”, “epigenomics”, “lung malignancy”, “lung adenocarcinoma”, “squamous cell carcinoma”, “WGS”, “WES”, “LPA”, “AIS”, and “MIA”.

For inclusion, articles were required to meet specific criteria: publication in the English language and use of integrative proteogenomic techniques. Articles reporting data from animal model studies and isolated genomic or proteomic studies were excluded from the present review process. The initial search yielded 58 articles. Following removal of duplicates, 55 studies remained, 20 of which were included in the present study after application of inclusion/exclusion criteria and final manual inspection.

The present article focuses on reviewing milestone studies that integrate proteogenomics platforms to elucidate features of lung cancer; therefore, the results of these studies will be presented according to sample and cancer type.

## 3. Results

### 3.1. Tissue Samples

#### 3.1.1. Non-Small Cell Lung Cancer—Adenocarcinoma

A study carried out by Biswas et al. in 2017 combined whole genome sequencing (WGS) with MS-proteomics to analyze the primary and metastatic tumor profile of a patient with metastatic lung adenocarcinoma receiving ERBB2-targeted therapy [27]. Interestingly, the compatibility of somatic mutations between the two tumor sites did not exceed 1%, while overly expressed ACTA2 molecules were deemed responsible for the early incidence of metastasis in this patient. Tumor cell proliferation was primarily attributed to activation of the ERBB2 and CDK12 pathways; lung and lymph node specific mutations were also detected. The above case-oriented analysis concluded that despite the presence of great tumor heterogeneity, key oncogenic mechanisms remain intact, as in the case of the patient studied.

The mechanisms driving intratumor mutational heterogeneity was explored by Roper et al. in their study of primary NSCLC [28]. They reported a total of between 182 and 1058 silent mutations in individual patient samples, identified using whole exome sequencing of either primary or metastatic tumors. According to this study, aberrations in *EGFR* and *KRAS* were encountered in both primary and metastatic tumors, while certain mutations such as *HRAS* could only be traced to metastatic tumors. Mutational tumor heterogeneity was among other parameters related to *TP53* mutation and significantly correlated with APOBEC mutagenesis, resulting from altered expression of APOBEC3 region transcripts. Overexpression of transcripts was linked to increased activity of interferon pathways [28].

In a combined proteomic, transcriptomic analysis of 51 surgically removed lung adenocarcinomas in 2018, Sharpnack et al. attempted to elucidate mechanisms driving disease relapse despite prompt and complete removal of the cancer tissue and the possible benefits of using adjuvant chemotherapy, or lack thereof [29]. The researchers used liquid chromatography-mass spectrometry, and RNA-sequencing in an effort to identify any biomarkers, quantifying both proteins and mRNA expression accordingly. These could be utilized toward better management of the disease with improvement of survival rate for patients, as they display a capacity for recurrence rate prediction. According to the study findings, translocase of inner mitochondrial membrane 50 (*TIMM50*), which encodes the protein Tim50, was the gene with the highest degree of differential correlation. Upregulation of Tim50, a protein of the mitochondrial apoptosis pathway resulting from mutant p53, has been demonstrated in cell lines [30].

Furthermore, in 2020, Nishimura et al. utilized mass spectrometry (MS) to identify and quantify a wide range of disease-related proteins which are specifically expressed in lung adenocarcinoma patients and are correlated with specific gene expression [31]. As suggested by the authors, protein identification may lead to appropriate categorization of adenocarcinoma specimens in the three relevant subtypes, i.e., lepidic predominant invasive adenocarcinoma (LPA), adenocarcinoma in situ (AIS), and minimally invasive adenocarcinoma (MIA). In turn, this may not simply improve disease prognosis but also assist in the correct treatment design, where these proteins can be associated with promising therapeutic targets. Most notably, tumor categorization in the above study into different subtypes was based on 2023 proteins [31].

In 2020, Chen et al. combined whole exome sequencing (WES), RNA-seq, proteomics, and phosphoproteomics to assess the proteogenomic profile of LUAD with a focus on East Asia, in a cohort mainly consisting of Taiwanese non-smokers (83%) [32]. High prevalence of *EGFR* mutations, with detection of these in 85% of patients, was revealed by genomic profiling of genes involved in oncogenesis according to the Cancer Gene Census (COSMIC). These were followed by *TP53* and *RBM10* mutations, occurring in 33% and 20% of patients respectively. Single nucleotide variations (SNVs) detected in this group of patients vastly differed from those reported in the Cancer Genome Atlas. The above discrepancy was not solely attributed to smoking, as both smokers and non-smokers in the examined cohort displayed similar proportions of SNVs. Similarly, a significantly higher frequency of several mutations was observed in the cohort of Chen et al. in comparison to other series. These included aberrations in *EGFR*, *RBM10* and *CDC27, RB1*, two genes involved in cell-cycle regulation. In contrast, the somatic mutations *TP53*, *KRAS*, and *KEAP1* displayed a lower frequency. Yet again, these conflicting findings were not artefacts generated by the lower percentage of smokers in this cohort, as isolated study of the non-smokers also revealed that *EGFR*, *RBM10*, *RNF213*, *ATP2B3*, and *TET2* mutations were many times more prevalent, while *KRAS* mutations were significantly lower. Hence, significant differences in the LUAD genomic profile of Taiwanese never-smokers compared to that registered in the Cancer Genome Atlas were discovered.

RNA sequencing and proteomic and phosphoproteomic analyses carried out on the same cohort revealed a transcriptional boost of DNA replication, glycolysis, glutathione pathways, and immune-associated processes. Moreover, proteomic data attested to upregulated DNA repair, protein refinement, transport mechanisms, and downregulated cell-adhesion processes. The proteogenomic layer of the above integrative analysis revealed a positive association of *TP53* mutations, with genes regulating the cell cycle and phosphoproteins modulating DNA topology and repair, providing further evidence of its established synthetic lethal functions [32]. The researchers noted co-modulation of the phosphorylation pattern in MAPK pathway components (proteins), which differentiated patients into those with high and those with low activation, the former being correlated with *EGFR* and *KRAS* mutations and the latter with *TP53* mutations, particularly in later stages. Hence, this evidence may shed light on the role of *TP53* in the regulation of key underlying mechanisms in non-small cell lung cancer (NSCLC).

Gillette et al. [33] undertook deep proteogenomic characterization of 110 lung adenocarcinoma (LUAD) tumors matched to 101 normal adjacent tissues (NAT). Their study employed a multi-omic approach, utilizing various acetylproteomic, phosphoproteomic, genomic, and epigenomic methods. Examined samples originated from the National Cancer Institute’s Clinical Proteomic Tumor Analysis Consortium (CPTAC), and the study was characterized by the inclusion of a more diverse population in terms of smoking status and ethnic diversity than previous research efforts in the field. Proteogenomic analysis revealed four distinct clusters within the LUAD tumor tissues examined: Cluster 1 (C1) was characterized as proximal-inflammatory, *STK11* wild-type, displaying an increased presence of *TP53* mutations and a high status of CpG island methylator phenotype (CIMP); Cluster 2 (C2), mainly stemming from patients of US origin, was identified as proximal-proliferative and characterized by wild-type *EGFR* and *TP53*, as well as an intermediate degree of CIMP; Cluster 3 (C3), also proximal-proliferative, was characterized by *STK11* mutations and predominantly included patients of Vietnamese nationality; Cluster 4 (C4), termed the terminal respiratory unit cluster, lacked *STK11* and *KRAS* mutations but exhibited a notable presence of *EGFR* mutations. This cluster mainly consisted of patients of Chinese nationality and female patients.

The abovementioned study revealed differential phosphorylation of ALK Y1507 in samples with *ALK* fusion, providing evidence of its importance in ALK fusion cancers. Observed extensive differences in the phosphoproteome highlighted potential druggable targets that could be modulated by kinase-targeting drugs. In other respects, Gillette et al. [33] discovered that that *STK11* mutations bestowed an immune-cold behavior, suggestive of an immune system escape mechanism through neutrophil degranulation. Furthermore, lung cancers retrieved from non-smokers tended to present a higher incidence of ARHGEF5 phosphorylation dysregulation, potentially indicating a druggable oncogenic mechanism. However, the interconnectedness of various factors such as mutation status, ethnic background, geographical location, gender, and smoking habits, which may introduce confounding factors, making the isolation of specific causal relationships challenging, was among the study limitations noted by the authors. Furthermore, the authors highlighted the lack of spatial and cellular resolution, which limited estimation of the microenvironment’s effect on tumor formation and development.

Clustering of surgically resected tumors from a US cohort consisting of 87 patients, based on their gene signature profiles, revealed the presence of three subtypes. Specifically, in their proteogenomic study, Soltis et al. [34] discovered a transition high, a transversion high, and a structurally altered subtype utilizing somatic genome signature analysis. Never-smokers with a high prevalence of *EGFR* mutations and current smokers with aberrations in *KRAS*, *STK11* composed the majority of the former two subtypes, respectively. A difference in TMB was noted regarding these subtypes, as this was very low in transition high and high in transversion high tumors. In contrast, the third subtype, termed structurally altered, was characterized by a high prevalence of former smokers, mutations in *TP53,* high TMB, and the overall highest degree of genetic alterations. For instance, the highest number of structural deletions and insertions were detected in tumor samples belonging to this subtype. The authors also explored RNA-protein correlation from a gene viewpoint. Remarkably, this was found to be associated with tumor purity, displaying a wider range in immune-enriched tumors. Predictors of clinical outcome, namely expression patterns of specific proteins, RNAs, and co-expressed RNAs-proteins, were identified by the study.

Proteogenomic clustering of tumors in Soltis et al. was performed according to previously defined RNA-based subtypes (Terminal Respiratory Unit—TRU, Proximal Proliferative—PP and Proximal Inflammatory—PI). Indeed, the authors detected high concordance between the RNA, protein, and multi-omic classification of tumors. Several targetable molecular pathways were detected within each subtype, requiring further validation. These included EGFR, IFN-γ signaling in TRU and PI subtype tumors respectively, and several modulations possibly responsive to CDK and glutaminase inhibitors in the PP subtype. Nevertheless, as admitted by the authors, the above study did not incorporate normal adjacent lung tissue samples.

Recently, a deletion in Chromosome 4, specifically that of Chr4q12, was found to drive adenocarcinoma progression from pre-invasive to invasive [35]. The molecular phenomena surrounding LUAD progression were investigated by Zhang et al. in an approximately equal number of pre-invasive and invasive tumors (98 and 99, respectively). Their proteogenomic investigation incorporated whole exon, RNA sequencing, proteomics, and phosphoproteomics. High prevalence of *TP53* mutations in IAC and the pre-existence of aberrations in *EGFR* in AIS/MIA subtype tumors were noted. Critically, the abovementioned chromosomal deletion was linked to a corresponding deletion in *SPATA18*, which, according to the authors, may promote tumor growth through mitophagy suppression. In conjunction with the above, the comprehensive proteomic analysis revealed the presence of three subtypes (SI, SII, SIII) representative of staging, with regard to tumor progression. The first subtype, primarily constituting of AIS/MIA subtype tumors, was associated with improved patient overall/recurrence-free survival. On the contrary, while SII acted as an intermediate subcategory, patients with IAC were mostly grouped in the SIII subtype, and worse patient overall/recurrence-free survival was observed for this group. However, the sole inclusion of East Asian patients in the study, thus resulting in the increased presence of non-smoking women and a high prevalence of *EGFR* mutations within the cohort, constituted major limitations, as stressed by the authors.

#### 3.1.2. Non-Small Cell Lung Cancer—Squamous Cell Carcinoma

A pilot proteogenomic study comparing LUAD to lung squamous cell carcinoma (LSCC) was conducted by Stewart et al. in 2015, using quantitative proteomics in conjunction with a customized AffymetrixGeneChip micro array platform [36]. Tissue samples used for the analysis were derived from three patients diagnosed with LUAD and three with LSCC, and the raw files required for comparative analyses were obtained from previously published data and/or the ProteomeXchange consortium database [36,37,38]. According to the results, differential expression of 565 proteins and 629 genes was discovered between LUAD and LSCC, while simultaneous gene and protein level differential expression was noted for 113 of these [36]. Overexpression of the proteins MCT1 (encoded by *SLC16A1*) and GLUT 1 (encoded by *SLC2A1*) in LSCC was noted by the authors in all examined studies; hence, these were investigated further. In combination with a survival analysis using publicly available datasets, the observed differential expression of these two proteins, with regard to the gene level, highlighted the role of MCT1 and GLUT1 in LUAD and LSCC as prognostic tools and therapeutic targets, respectively [36].

In a study of 108 patients, most of whom were of non-Hispanic white ethnicity/race, Stewart et al. integrated mass spectrometry with RNA-seq and genomic analysis in an attempt to define LSCC molecular subtypes and identify major alterations that drive disease pathogenesis [39]. Based on proteomic characterization, three subtypes were identified, with the first two encompassing the majority of tumor samples (87%). In detail, these included an Inflamed and a Redox subtype, the former associated with immune cell infiltration and the latter enriched for oxidation-reduction pathways [39]. Hence, according to the authors, therapies targeting immune cells, including neutrophils and/or B cells, or the metabolic modulation of tumor intrinsic pathways could prove beneficial for LSCC patients. Critically, the Inflamed subtype exhibited elevated expression of *PD-1*, whereas more NFE2L2/KEAP1 alterations and copy gain in 3q2 locus were found in Redox subtype tumors. Concerning this subtype, metabolic vulnerabilities associated with *TP63*, *PSAT1*, and *TFRC* were identified. Whilst Stewart et al. noted a lack of correlation between the above proteomic subtypes and patient survival, they also discovered that the presence of B-cell-enriched lymph nodes, commonly found among the Inflamed cluster, conferred better survival. According to the authors, compared to previous research efforts [23,38,40], their study constituted the most comprehensive integrative analysis of genomic, transcriptomic, and proteomic datasets in lung cancer [36,37,41].

Satpathy et al. in 2022 analyzed 108 prospectively collected treatment-naïve primary lung squamous cell carcinoma (LSCC) tumors and 99 paired normal adjacent tissues (NATs) [42]. Their study focused on the discovery of druggable therapeutic protein targets, additionally investigating cellular signaling pathways and exploring post-translational modifications. A total of 5523 copy number alteration-mRNA events were observed, 2154 of which displayed significant correlation with protein expression. Among the latter, 138 cancer-associated genes were identified. The discovery of six amplified (i.e., *WHSC1L1*, *CCND1*, and *SOX2*) and 29 deleted (i.e., *NCOR1*, *SETD2*, and *CBL*) cancer associated genes was highlighted. Clustering based on the genomic, transcriptomic, and proteomic data obtained from the respective analyses of the 108 tumor tissues revealed five molecular subtypes of LSCC. The first subtype, termed “Basal-Inclusive”—(B-I), exhibited metabolic, immune, and estrogen receptor signaling. EMT-related pathways were found to be upregulated in the second subtype (“Epithelial to Mesenchymal Transition-Enriched”—EMT-E), which further displayed phosphorylation-driven PDGFR and ROR2 signaling. The third subtype (“Classical”) was characterized by mutations in *CUL3*, *KEAP1*, and *NFE2L2*, along with high-level amplification of *SOX2* and *TP63.* In the fourth subtype (“Inflamed-Secretory” (I-S)) immune-related pathways were observed as being significantly upregulated. Finally, the fifth subtype (“Proliferative-Primitive” (P-P)) exhibited upregulation of proliferation-related pathways, downregulation of immune signaling, and enrichment of CIMP-low samples. Remarkably, all subtypes displayed a loss of CDK4/6 pathway inhibitors, while the expression of Rb1 displayed great variability amongst subtypes. However, as noted by the authors, their study [42] was constrained by inherent tumor heterogeneity.

#### 3.1.3. Non-Small Cell Lung Cancer—All Subtypes

Lehtiö et al. [43] conducted a deep proteogenomic characterization of 141 non-small cell lung cancer (NSCLC) tumors, encompassing all major histological groups. Approximately 14,000 proteins were examined, providing an overview of the molecular landscape of NSCLC. Their study identified six distinct subtypes of NSCLC tumors: Subtype 1 was characterized by a comparatively higher occurrence of *EGFR* mutations. Meanwhile, subtypes 2 and 3 were distinguished by significant immune infiltration levels, an observation reflected in the expression of neoantigens and chemokines, thereby suggesting an active immune response among these. On the other hand, Subtype 4 exhibited mutations in *KEAP1*, *STK11*, and *SMARCA4.* Intriguingly, the highest count of overexpressed oncogenes per sample displaying similar overexpression of RET receptor tyrosine kinase was observed in this subtype. The majority of samples in Subtype 5 consisted of large cell neuroendocrine carcinoma (LCNEC) tumors. Subtype 5 exhibited a higher occurrence of *RB1* mutations and demonstrated overexpression of the transcriptional activator MYB, as well as the proteins BCL2 and CDK2. Additionally, it displayed increased E2F signaling, which was possibly attributed to reduced degradation of E2F1. Subtype 6 was primarily comprised of squamous cell carcinoma (SqCC) cells. This subtype was characterized by *TP53* mutations and elevated expression of the surface protein B7-H4. Increased expression of B7-H4 has been additionally implicated in reduced immune response against these tumors [44].

A phosphoproteomic investigation of extracellular vesicles by Qiao et al. in 2022 attempted to elucidate the role of kinase networks in lung cancer. Specimens from 13 subjects with NSCLC were analyzed and 1567 proteins harboring 2473 phosphorylation sites were identified. Regarding the kinase network, 152 kinases were recognized, 25 of which had expression alterations. Key phosphoproteins of this study included MAPK6S189, IKBKES172, SRCY530, CDK7S164, and CDK1 [25].

#### 3.1.4. Small Cell Lung Cancer

Despite the high incidence of mortality associated with SCLC, the proteogenomic landscape of this lung cancer type remains, for the most part, unexplored. In their recent study of 112 tumors and paired normal adjacent tissues, Liu et al. [45] attempted to delineate the disease proteogenomic features and identify prognostic markers. Additionally, the discovery of treatment strategies adapted to specific tumor subtypes was attempted. The authors demonstrated a correlation between multiple genetic alterations, such as aberrant expression of *FAT1*, deletion of *RB1*, loss of the long (q) arm of chromosome 5, and lung cancer. The proteomic analysis yielded 138 proteins with at least twofold change in expression, 25 of which were present in the majority (90%) of tumor-NAT pairs. Moreover, two novel biomarkers with prognostic value were discovered and experimentally validated by Liu et al., namely the proteins HMGB3 and CASP10. As noted by the authors, patient overall survival was associated with protein expression levels: overexpression of HMGB3 resulted in poor outcomes, with the opposite being true for CASP10. Furthermore, immune cell infiltration was correlated with *ZFHX3* mutation and, according to the proteogenomic analysis, DDR activity was linked to immune suppression via dissipation of cGAS-STING pathway activation. Finally, unsupervised clustering was utilized to divide tumors into four subtypes with distinct biological characteristics and therefore unique treatment vulnerabilities. Nevertheless, Liu et al. point towards the need for a concerted effort to validate conclusions reached by the above study, as a significant fraction of the identified molecular aberrations were hypothesis-generating.

Therefore, it becomes evident that proteogenomic analysis of clinical samples offers a unique insight into the complex mechanisms governing lung cancer. The inclusion of diverse patient cohorts in proteogenomics studies has revealed distinct patterns of genetic aberrations (Table 1).

Furthermore, this plethora of genomic information, analyzed in tandem with generated patient proteomic profiles and gene expression data, has enabled the discovery of novel drug targets and prognostic markers (Table 2). Correlation analyses of mutations in genes and identified proteins have been presented by relevant studies [25,33,34,35,42]. For instance, Gillette et al. [33] observed a correlation between *RB1* mutation and CDK4 protein abundance and *EGFR* mutation with CTNNB1 protein expression. Meanwhile, Satpathy et al. [42] linked *RB1* mutations to expression patterns of cell cycle proteins. It is noteworthy that proteogenomic clustering has unearthed unexplored associations between mutations and proteins [34].

### 3.2. Cell Lines

Treue et al. [46] presented a systems analysis of cell lines in a model of EGFR-mutated non-small cell lung cancer (NSCLC) resistant to targeted therapy, the aim being to identify novel mechanisms of resistance and propose combination therapies. The analysis constituted an integration of mass spectrometry-based discovery time-course phosphoproteomics with whole exome sequencing and computational modeling. In more detail, this approach was applied to the H1650 and HCC827 human lung cancer cell lines that displayed different responsiveness to gefitinib, an EGFR tyrosine kinase inhibitor, due to an EGFR Exon 19 mutation. The global phosphoproteomic changes were analyzed in a time-resolved manner in order to identify mechanisms of targeted therapy resistance and relate the phosphoprotein profiles to the corresponding mutational ones [46].

In total, 44 proteins and 35 topologically close genetic alterations were discovered after the authors successfully reduced the complexity of 2186 genetic variants, including 1312 copy number variations and 873 simple somatic mutations [46]. Interestingly, single and/or combination drug testing against the predicted phosphoproteins revealed that exploitation of HSBP1, DBNL, and AKT1 as therapeutic targets ultimately inhibits cell proliferation, thus surmounting resistance against EGFR inhibitors [46]. Despite the fact that the results of the study demonstrated an efficacy in overcoming resistance to EGFR inhibition, poor availability of inhibitors against some of the predicted phosphosite targets, as well as the use of indirectly acting (such as thiolutin for HSBP1) and/or of small-molecule inhibitors characterized by various off-target effects, served as strong limitations of the research conducted [46].

In a study by Wu et al., 14 differentially expressed proteins were found among non-small cell lung cancer (NSCLC) and small cell lung cancer (SCLC) cell lines, using quantitative proteomic analysis and transcriptomic data [47]. These included annexin A1(ANXA1) and annexin A2 (ANXA2), both known to be associated with lung cancer. According to the bioinformatic analysis conducted, all 14 factors were implicated in disease modulation, as they were strongly involved in the proliferation, migration, and invasion of lung cancer cells. It is noteworthy that a positive correlation was discovered between these proteins and their gene expression data, while, in some cases, a correlation with lung cancer was established for the first time. For the quantitative proteomics-based comparative analysis, cell lines representative of each lung cancer type were used, namely A549 and H1975, for the study of NSCLC, and H446, H69 for SCLC [47]. Proteomic analysis identified a total of 3970 proteins, 147 of which were differentially expressed. A major limitation of the Wu et al. study, as highlighted by the authors, was the use of cell lines rather than cancer tissue samples.

Proteogenomic analysis, primarily of cell lines, yielded promising results with regard to both high tumor mutational burden (TMB) and low tumor mutational burden (TMB) cancers. Qi et al. [48] used a combination of NGS and mass spectrometry to identify proteins prevalent in melanoma and lung adenocarcinoma, with the ultimate goal of identifying immunogenic human leukocyte antigen (HLA) class I-presented peptides. Twelve variant peptides and 40 class I-presented CG antigen-derived peptides were discovered; according to the researchers, these could prove beneficial in vaccine and general precision immunotherapy development and could be used as treatments in other types of cancer [48].

### 3.3. Bioinformatic Analyses of Retrieved Multi-Omics Data

N^6^-methyladenosine (m^6^A) is a ribonucleic acid modification implicated in oncogenesis [49]. The clinicopathological significance and multi-omic profile of m6-A-related genes in LUAD diagnosis and prognosis were investigated by Wang et al. [50]. RNA-sequencing data was obtained from the Cancer Genome Atlas (TCGA) database, and a set of 21 previously identified m6A regulators were analyzed. Univariate Cox regression was used to determine the correlation between overall survival (OS) of LUAD patients and m6A-linked gene transcription, and a patient risk profile was constructed using the LASSO (least absolute shrinkage and selection operator) approach [50]. Based on analysis of the transcriptomic data, 18 m6A regulators were significantly differentially expressed in LUAD tumors, a third (6) of which were associated with patient overall survival. Interestingly, the IGF2BP1, IGF2BP2, and HNRNPC genes displayed strong prognostic performance in adenocarcinoma, as validated in two independent cohorts of patients [50]. Regarding the risk profiles, the authors noted that a high-risk score was suggestive of drug resistance, presence of *TP53* mutation, and increased tumor immune cell infiltration. Wang et al. [50] validated *HNRNPC* involvement in cell proliferation and/or invasion in vitro in a cell culture analysis and transwell assay utilizing cell lines, transfected so as to modulate (up/ down regulate) *HNRNPC* expression. Hence, a novel perspective, with regard to the prognostic value of gene expression and personalized medicine for lung adenocarcinoma, was described [50].

Regulation of signaling pathways, including those involved in cytolysis, by the immunomodulatory factor TIM3 may modulate the proliferative ability and cell infiltration of lung adenocarcinoma cells, according to observations by Wu et al. [51]. It is also suggested that the above factor may control the tumor immune microenvironment in LUAD. Notably, an indirect influence on predicted patient outcomes and survival was discovered, arising from risk correlation with tumor cell resistance to therapeutic agents. In their data-oriented multi-omic analysis, the researchers utilized Cox and LASSO regression to filter genes related to cytolytic activity. Overall, differential expression of 450 genes associated with cytolysis was revealed, 273 of which were upregulated and 177 downregulated. Predictive value, with regard to the course of disease in LUAD, was ascertained for 91 genes. Moreover, Kaplan Meir survival curves were constructed for patients respectively identified as high and low risk through prognostic modeling. The generated risk score was also associated with patient sensitivity to the therapeutic agents AKT inhibitor VIII, Lenalidomide, and Tipifarnib [51]. Immunohistochemistry studies were carried out on 10 lung cancer tissues in order to characterize the expression of key immunomodulatory factors, with a focus on TIM3. The high expression of TIM3 in low-risk patients points to the need for further elucidation of its role as a regulator in cytolysis, especially within the context of novel drug target exploration.

Further, it is worth noting that several chromosomal aberrations have been correlated with multiple types of malignancies. In particular, genetic mutations located on Chromosome 9 (Chr9) have been implicated in the emergence of various cancer types, including lung cancer [52,53]. Proteogenomic data was analyzed by Ahn et al. [54] in order to pinpoint Chromosome 9 proteins (Chr 9), SNPs, and mutations involved in lung cancer and to characterize Chr9-encoded “missing proteins”. Missing proteins are those whose existence is supported by genetic evidence, but which have not yet been verified through mass spectrometry (MS) or antibody detection [55]. Their study detected 15 Chr9 proteins highly selective for lung cancer in comparison to healthy lung tissue (i.e., RAD23B, RPS6, ARPC5). Interestingly, correlation with various cancer types had previously been established for the majority of these, with some having been directly associated with lung cancer. For instance, mutated variants of the UV excision repair protein (RAD23B) were linked to the emergence of primary lung cancer regardless of patient ethnicity, and RPS6 was associated with drug resistance in non-small cell lung cancer, whereas the protein ARPC5 was found to upregulate a tumor suppressor agent in lung squamous cell carcinoma [56,57,58]. Additionally, the researchers discovered four peptides identified in lung cancer cells, which had amino acid substitutions owing to known mutations of their encoding genes, therefore demonstrating a link between proteomic analysis and registered genomic data [54].

## 4. Discussion

**Figure 1:**
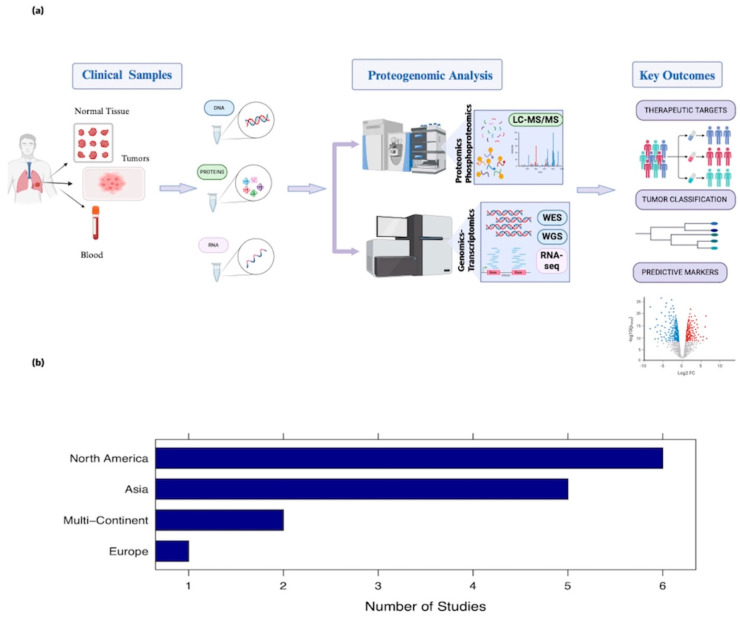
Overview of lung cancer proteogenomic studies. (**a**) Schematic representation of a proteogenomics workflow commonly adopted by the majority of investigated articles. (**b**) Boxplot of geographic distribution of patient population cohorts by continent (only studies with adequate data availability were included). Multi − Continent: North America, Europe, and Asia.

Lung cancer is a heterogenous disease affecting patients from diverse backgrounds and is associated with a high incidence of mortality [5]. The aim of this review article was to provide a comprehensive synthesis of landmark proteogenomic studies, thus highlighting the potential of this innovative platform as a tool of anticancer research. While molecular mechanisms governing crucial cancer traits, e.g., neoangiogenesis, apoptosis resistance, immune evasion, and metastatic potential, have been previously addressed by genomics, proteogenomics is anticipated to fill significant knowledge gaps and assist in the discovery of novel treatment options (Figure 1). As echoed in our findings, present research efforts reflect disease epidemiology, primarily focusing on adenocarcinoma, exploring metastatic potential, cancer progression, and hallmarks in non-smokers. It is worth noting that patients from North America are the most frequently studied cohort to date, followed by Asian non-smokers (Figure 1).

**Figure 2:**
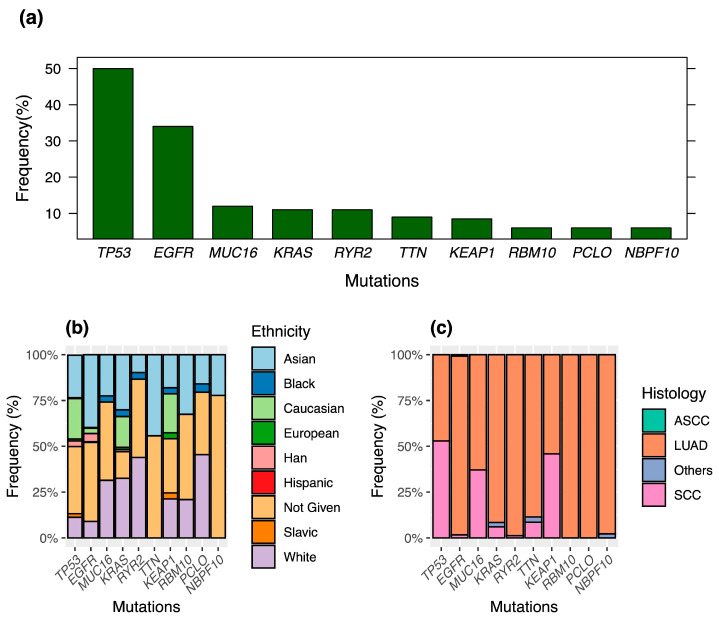
Mutational landscape of patient cohorts from reviewed articles. (**a**) Top 10 driver mutations based on frequency (%) of appearance in the examined cohorts *. (**b**,**c**) Relative incidence of these top 10 driver mutations in different ethnicities and lung cancer types, as defined by histological evidence. * Mutation data were publicly available in 5 studies, involving a total of 716 patients. Han: Han Chinese; ASCC: Adenosquamous Carcinoma; LUAD: Lung Adenocarcinoma; SCC: Squamous Cell Carcinoma.

Genomic profiling provides evidence of shared driver mutations among patients, including *EGFR*, *KRAS*, *RBM10*, *TP53*, and other genes (as seen in Table 1). In particular, the mutational landscape driving adenocarcinoma incidence in East Asia has been linked to *EGFR* mutations and patient demographics, including age and sex [32]. Mutation frequencies and relative incidence among patients of different ethnicities and cancer types is presented in Figure 2.

**Figure 3:**
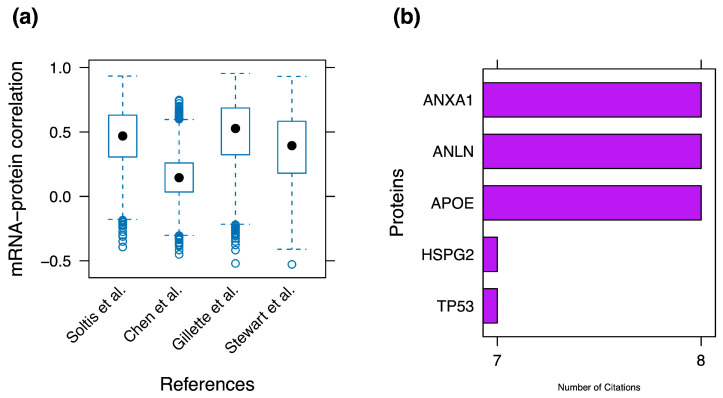
Key proteogenomic findings derived from reviewed articles. (**a**) Boxplot depicting gene-wise mRNA-protein correlation in tumor samples of cohorts corresponding to the references on the x-axis [32,33,34,39]. Circles represent outlier and central (black) dots represent median values. The y-axis values represent mRNA-protein correlation expressed as Spearman’s correlation. (**b**) Incidence of top 5 differentially expressed proteins in the examined paper cohort. Only studies with adequate data availability were included.

Nonetheless, gene aberrance is characterized by greater diversity. Exploration of the downstream effect generated by genomic alterations has uncovered great variability in gene- and sample-wise mRNA-protein correlation (Figure 3). Differential expression of genes may not be concurrently reflected in transcripts and proteins, and could be tissue-specific with regard to the latter [28,29] (Figure 3). Besides being an indicator of cellular process regulation, mRNA-protein correlation is a valuable prognostic tool in the discovery of biomarkers indicative of lung cancer recurrence [29].

Much emphasis has been placed on defining proteogenomic subtypes of tumors belonging to the major histological classes. Multi-omic data integrative analysis has been utilized to cluster patient samples displaying unique mutational gene expression patterns and proteomic, phosphoproteomic profiles. Proteogenomic clusters highlight distinct therapeutic vulnerabilities displayed by tumors, aiding the discovery of novel drug targets [32,33,34,35,42,45]. Therefore, a diverse picture of therapeutic targets has emerged, including metaloproteinases (e.g., MMP2, MMP11, MMP2, MMP4) significantly upregulated in East Asian non-smokers with LUAD [32], the proteins MCT1 and GLUT1 overexpressed in LUAD [36] and SOS, and PTPN11/Sph2 targetable in patients harboring *KRAS* and *EGFR* mutations [33], to name a few (Table 2). This is of particular significance, as very few of these have either been characterized to date or are entirely lacking in well-defined, i.e., LUAD, and rare, i.e., SCLC, lung cancer types, respectively [33,45]. Additionally, a similar landscape of prognostic markers in NSCLC has arisen, while proteogenomic characterization of SCLC is in its infancy.

Furthermore, valuable insights have been generated by the multi-omic analysis of publicly available omics data, such as those retrievable from the Cancer Genome Atlas portal. Specifically, integrative bioinformatic analysis of genomics, transcriptomics, and proteomics data has aided in the discovery of prognostic markers, including the protein TIM3 and the gene *HNRNPC* [50,51], while few studies have utilized cell lines to explore the proteogenomic landscape of lung cancer, yielding significant findings awaiting validation in clinical samples [46,47,48].

It is, therefore, evident that the application of proteogenomics in lung cancer has already generated valuable and potentially actionable information. Future studies should aim to include larger and more diverse patient cohorts, reflecting disease prevalence. This would enable further delineation of molecular mechanisms governing lung cancer and their downstream effects.

## 5. Conclusions

While a “unified theory” of oncogenesis and cancer progression is likely elusive due to the complexities of these processes, there is hope that clinically translatable answers may arise. These answers would deal with specific subtypes of cancer or act as guidance for personalized patient care. Extensive and detailed mapping in fields where knowledge is lacking can be leveraged against cancer, including the emergence of key driver mutations or the deeper ramifications of perturbations in molecular networks underlying the disease [59]. This will bring us a step closer to the application of personalized medicine for the treatment of lung cancer.

## Figures and Tables

**Table 1:** Characteristics of proteogenomics studies utilizing clinical samples.

First Author & Year of Publication	Study Design	Region & Period of Sample Collection	Age (Years)	Cancer Type	Aberrantly Expressed Genes	Driver Mutations
Biswas et al., 2017 [27]	NA	North America NA	NA	LUAD	*ACTA2*	*CDK12* G879V
Roper et al., 2019 [28]	SC	North America December 2013–Present	42–71	LUAD, TC	*CCND1*, *STAT1*	*EGFR*, *KRAS*, *HRAS*, *TP53*, *STK11*, *CLYB*, *CREBBP*, *CTNNB1*, *EML4*, *EXT2*, *FOXL2*, *GNAS*, *MED12*, *MLL3*, *MTOR*, *NTRK1*, *PTPRD*, *RICTOR*, *STAT3*, *EGFRT790M*, etc.
Sharpnack et al., 2008 [29]	MC	North America NA	NA	LUAD	*TIMM50* (325 hypotheses of dysregulated genes)	NA
Nishimura et al., 2020 [31]	SC	Asia 2000–2014	53–78	LUAD (AIS, MIA, LPA)	NA	*EGFR*, *ERBB2* p. Gly776delinValVal
Chen et al., 2020 [32]	MC	Asia July 2016–July 2018	40–86	LUAD, LSCC, ASC, Other	*RBM10*	*EGFR*, *TTN*, *TP53*, *RBM10*, *KRAS*, *RNF13*, *MUC4*, *MUC15*, *FLG*, etc.
Gillette et al., 2020 [27]	MC	North America, Asia, Europe 2011–Present	35–81	LUAD	*CALU*, *CIAO1*, *PRPF40B*, *PLEC*, *MUC22*, etc..	*KRAS*, *EGFR*, *ALK*, *RB1*, *KEAP1*, *STK11*, *TP53* etc.
Soltis et al., 2022 [34]	MC	North America 2012–2018	41–85	LUAD	NA	*TP53*, *KRAS*, *STK11*, *EGFR*, *TLR4*, *KEAP1*, *RBM10*, *PIK3CA*, *SFTPB 3′ UTR*, *ZNHIT6 prom*., etc.
Zhang et al., 2024 [35]	SC	Asia NA	28–84	LUAD (AIS, MIA, IAC)	*SPATA18*, *NPC1*, *TIMM17B*, *NDUFA1 EGFR* etc.	*EGFR*, *TP53*, *MUC16*, *TTN*, *RBM10*, *RYR2*, *ERBB2*, *BRAF*, etc.
Stewart et al., 2015 [36]	SC	North America NA	NA	LUAD, LSCC	*MCT1*, *GLUT1*, *LMO7* etc. (629 differentially expressed genes)	NA
Stewart et al., 2019 [39]	SC	North America NA	63–78	LSCC	*PD-1*	*TP53*, *MLL22*, *NFE2L2*, *KEAP1*, *RB1*, *APC*, *CTNNB1* etc.
Satpathy et al., 2021 [42]	MC	North America, Asia, Europe May 2016–August 2018	40–88	LSCC	*LRIF1*, *PITX1*, *REPIN1*, *TRPS1*, *PLAU*, *FADS2*, *PTGS3*, *RPP25*, *ZNF597*, *SN16A3*, *MTCL1*, *FAM110A*, *MX1*, *FAM50A* etc.	*TP53*, *ARID1A*, *KMT2D*, *CDKN2A*, *CUL3*, *KEAP1*, *NFE2L2*, *PTEN*, *NF1*, etc.
Lehtiö et al., 2021 [43]	MC	Europe Pre–2004/2014–present/2006–2015	36–84	NSCLC (all major histological types)	*HNF1A*, *FGL1*, *CPS1* (speculated)	*EGFR*, *STK11*, *KEAP1*, *SMARCA4*, *RB1*, *TP53*, *KRAS*
Qiao et al., 2022 [25]	SC	Asia NA	55 ± 8, 55 ± 10	LUAD, LSCC	NA	NA
Liu et al., 2024 [45]	SC	Asia April 2012–June 2019	38–81	SCLC	NA	*TP53*, *GNAS*, *FAT1*

LUAD: Lung Adenocarcinoma; TC: Thymic Carcinoma; AIS: Adenocarcinoma In Situ; MIA: Minimally Invasive Adenocarcinoma; LPA: Lepidic-predominant Adenocarcinoma; LSCC: Lung Squamous Cell Carcinoma; ASC: Adenosquamous Carcinoma; IAC: Invasive Adenocarcinoma; NSCLC: Non-Small Cell Lung Cancer; SCLC: Small Cell Lung Cancer. SC: Single Center, MC: Multicenter.

**Table 2:** Proteogenomic findings and key outcomes of reviewed studies investigating clinical samples.

First Author & Year of Publication	Cancer Type	Proteins Differentially Expressed	mRNA-Protein Correlation (Spearman’s ρ)	Therapeutic Targets	Prognostic Markers
Sharpnack et al., 2018 [29]	LUAD	NA (N = 66 differentially expressed proteins)	0.07/0.017 (*)	NA	*SUMO1*, *PCBD1*, *PSMC5*, *ARCN1*, *PPA2*, *SRI*
Nishimura et al., 2020 [31]	LUAD (AIS, MIA, LPA)	SRPRB, HYOU1	NA	NA	NA
Chen et al., 2020 [32]	LUAD, LSCC, ASC, Other	NSCLC pathway: RBM10, EGFR, ERBB2, Ras, PCK, JAK3, STATs etc. Other: AKT1, ADAMTS4, AHCY, AKR1A1, AKR1C3	0.14 (0.31) (#)	MMP2, MMP11, MMP12, MMP14, etc.	MMP11
Gillette et al., 2020 [33]	LUAD	GREM1, LAPTM4A, GFPT1, BZW2, PDIA4, P4HB, PMM2, CDK1, CCNB1, MET, CXL8, THY1, etc.	0.53 (0.525) (#)	PTPN11/Shp2, SOS1, STK11	ERO1A, DHFR, MANF, HYOU1, LDHA, CBX8
Soltis et al., 2022 [34]	LUAD	IRS2, PKM, NIT2, GSTP1, GSR, CBR1, GPX2, GCLM, GCLC, CPS1, GPT2 PFAS, CTPS2, PPAT, CTPS1, GMPS, GFPT1, ADSSL1, GDAP1, GPX4, GPX1, GLS, SDC, LPCAT3, SCD, HELLS, G6PD, CYP4F11, AKR1C3, AKR1C2	0.47 (0.23–0.69) (#)	PD-L1, PRKCE, RPS6KA1	SAMA4B, ERO1A, MAFF, GAPDHS, CREG1, RFXAP, CCT8, etc.
Zhang et al., 2024 [35]	LUAD (AIS, MIA, IAC)	SPATA18, NPC2, VPS11, CFD, FCN3, C2, C5, C6, C7, C8B, APOH, SDR16C5, CARDS2, LOX TAOK3, etc.	0.39	NA	SPATA18, TIMM17B, GHITM, LAMC2, CHDH5, CFB, C2, APOH, SDR16, C5, CARDS2, LOX, TAOK3, STX4, NOTCH1, C1QB, SERPINA1, CDK7
Stewart et al., 2015 [36]	LUAD, LSCC	KRT6C, KRT6A, KRT6B, PKP1, MCT1, COL7A1, GLUT1, ABCF3, LMO7 ^1^	0.16	MCT1, GLUT1 (in LSCC only)	MCT1, GLUT1 (in LUAD)
Stewart et al., 2019 [39]	LSCC	NA	0.38	PSAT1, TP63, TFRC	Presence/Absence of TLN
Satpathy et al., 2021 [42]	LSCC	TGFBR2, MSI2, SPRED1, SF3B1, SESN1, UBR5, CDKN2C, IFNGR1, NUF2, CDKN1B, MSI1, SLFN11, etc.	NA	NSD3, BIRC5, LSD1, KDM3A, EZH2	TOP2A, ZC3H8, CDCA8, SMC2, QSOX2, HSPA5
Qiao et al., 2022 [35]	LUAD, LSCC	SCR, MAPK6, CDK1, CDK7	NA	BUB1, CAV1, CDK3, ERBB3, MAP2K4, MAP3K5, MAP3K8, PTK7, PTPN6, STAM, TRIM24	NA
Liu et al., 2024 [45]	SCLC	STMN2, STMN1, UCLH1, H1-5, TOP2A, TMA7, FEN1, MCM6, PCNA, MCM4, MCM3, MCM7, PARP1, etc.	0.47 (0.31)	ATR, TOP1, DLL3	HMGB3, CASP10

* Measured in two cohorts (comparisons of 3004 and 4656 genes, respectively); # Gene-wise and sample-wise correlation in (); ^1^ Differential protein expression was investigated between LUAD and LSCC.

## References

[1] Sung H., Ferlay J., Siegel R.L., Laversanne M., Soerjomataram I., Jemal A., Bray F. (2021). Global Cancer Statistics 2020: GLOBOCAN Estimates of Incidence and Mortality Worldwide for 36 Cancers in 185 Countries. CA. Cancer J. Clin..

[2] Jeon D.S., Kim H.C., Kim S.H., Kim T.-J., Kim H.K., Moon M.H., Beck K.S., Suh Y.-G., Song C., Ahn J.S. (2023). Five-Year Overall Survival and Prognostic Factors in Patients with Lung Cancer: Results from the Korean Association of Lung Cancer Registry (KALC-R) 2015. Cancer Res. Treat..

[3] He S., Li H., Cao M., Sun D., Yang F., Yan X., Zhang S., He Y., Du L., Sun X. (2022). Survival of 7,311 Lung Cancer Patients by Pathological Stage and Histological Classification: A Multicenter Hospital-Based Study in China. Transl. Lung Cancer Res..

[4] Siegel R.L., Miller K.D., Fuchs H.E., Jemal A. (2021). Cancer Statistics, 2021. CA Cancer J. Clin..

[5] Leiter A., Veluswamy R.R., Wisnivesky J.P. (2023). The Global Burden of Lung Cancer: Current Status and Future Trends. Nat. Rev. Clin. Oncol..

[6] Travis W.D., Brambilla E., Nicholson A.G., Yatabe Y., Austin J.H.M., Beasley M.B., Chirieac L.R., Dacic S., Duhig E., Flieder D.B. (2015). The 2015 World Health Organization Classification of Lung Tumors. J. Thorac. Oncol..

[7] Testa U., Castelli G., Pelosi E. (2018). Lung Cancers: Molecular Characterization, Clonal Heterogeneity and Evolution, and Cancer Stem Cells. Cancers.

[8] Thun M., Peto R., Boreham J., Lopez A.D. (2012). Stages of the Cigarette Epidemic on Entering Its Second Century. Tob. Control.

[9] Sun S., Schiller J.H., Gazdar A.F. (2007). Lung Cancer in Never Smokers—A Different Disease. Nat. Rev. Cancer.

[10] Bach P.B. (2009). Smoking as a Factor in Causing Lung Cancer. JAMA.

[11] Zhou F., Zhou C. (2018). Lung Cancer in Never Smokers—The East Asian Experience. Transl. Lung Cancer Res..

[12] Toh C.-K., Wong E.-H., Lim W.-T., Leong S.-S., Fong K.-W., Wee J., Tan E.-H. (2004). The Impact of Smoking Status on the Behavior and Survival Outcome of Patients with Advanced Non-Small Cell Lung Cancer. Chest.

[13] Chen T.-Y., Fang Y.-H., Chen H.-L., Chang C.-H., Huang H., Chen Y.-S., Liao K.-M., Wu H.-Y., Chang G.-C., Tsai Y.-H. (2020). Impact of Cooking Oil Fume Exposure and Fume Extractor Use on Lung Cancer Risk in Non-Smoking Han Chinese Women. Sci. Rep..

[14] Barone-Adesi F., Chapman R.S., Silverman D.T., He X., Hu W., Vermeulen R., Ning B., Fraumeni J.F., Rothman N., Lan Q. (2012). Risk of Lung Cancer Associated with Domestic Use of Coal in Xuanwei, China: Retrospective Cohort Study. BMJ.

[15] Kurmi O.P., Arya P.H., Lam K.-B.H., Sorahan T., Ayres J.G. (2012). Lung Cancer Risk and Solid Fuel Smoke Exposure: A Systematic Review and Meta-Analysis. Eur. Respir. J..

[16] Tseng C.-H., Tsuang B.-J., Chiang C.-J., Ku K.-C., Tseng J.-S., Yang T.-Y., Hsu K.-H., Chen K.-C., Yu S.-L., Lee W.-C. (2019). The Relationship Between Air Pollution and Lung Cancer in Nonsmokers in Taiwan. J. Thorac. Oncol..

[17] Samet J.M., Avila-Tang E., Boffetta P., Hannan L.M., Olivo-Marston S., Thun M.J., Rudin C.M. (2009). Lung Cancer in Never Smokers: Clinical Epidemiology and Environmental Risk Factors. Clin. Cancer Res..

[18] Seow W.J., Matsuo K., Hsiung C.A., Shiraishi K., Song M., Kim H.N., Wong M.P., Hong Y.-C., Hosgood H.D., Wang Z. (2016). Association between GWAS-Identified Lung Adenocarcinoma Susceptibility Loci and *EGFR* Mutations in Never-Smoking Asian Women, and Comparison with Findings from Western Populations. Hum. Mol. Genet..

[19] LoPiccolo J., Gusev A., Christiani D.C., Jänne P.A. (2024). Lung Cancer in Patients Who Have Never Smoked—An Emerging Disease. Nat. Rev. Clin. Oncol..

[20] Anagnostopoulos A.K., Gaitanis A., Gkiozos I., Athanasiadis E.I., Chatziioannou S.N., Syrigos K.N., Thanos D., Chatziioannou A.N., Papanikolaou N. (2022). Radiomics/Radiogenomics in Lung Cancer: Basic Principles and Initial Clinical Results. Cancers.

[21] Akhoundova D., Rubin M.A. (2022). Clinical Application of Advanced Multi-Omics Tumor Profiling: Shaping Precision Oncology of the Future. Cancer Cell.

[22] Campbell J.D., Alexandrov A., Kim J., Wala J., Berger A.H., Pedamallu C.S., Shukla S.A., Guo G., Brooks A.N., Murray B.A. (2016). Distinct Patterns of Somatic Genome Alterations in Lung Adenocarcinomas and Squamous Cell Carcinomas. Nat. Genet..

[23] (2014). Comprehensive Molecular Profiling of Lung Adenocarcinoma. Nature.

[24] Chen J.-Y., Chou H.-H., Lim S.C., Huang Y.-J., Lai K.-C., Guo C.-L., Tung C.-Y., Su C.-T., Wang J., Liu E. (2022). Multiomic Characterization and Drug Testing Establish Circulating Tumor Cells as an Ex Vivo Tool for Personalized Medicine. iScience.

[25] Qiao Z., Kong Y., Zhang Y., Qian L., Wang Z., Guan X., Lu H., Xiao H. (2022). Phosphoproteomics of Extracellular Vesicles Integrated with Multiomics Analysis Reveals Novel Kinase Networks for Lung Cancer. Mol. Carcinog..

[26] Page M.J., McKenzie J.E., Bossuyt P.M., Boutron I., Hoffmann T.C., Mulrow C.D., Shamseer L., Tetzlaff J.M., Akl E.A., Brennan S.E. (2021). The PRISMA 2020 Statement: An Updated Guideline for Reporting Systematic Reviews. BMJ.

[27] Biswas R., Gao S., Cultraro C., Zhang X., Maity T., Carter C., Thomas A., Rajan A., Hanada K.-I., Song Y. (2017). P2.01-041 Integrated Proteo-Genomics Analyses Reveal Extensive Tumor Heterogeneity and Novel Somatic Variants in Lung Adenocarcinoma. J. Thorac. Oncol..

[28] Roper N., Gao S., Maity T.K., Banday A.R., Zhang X., Venugopalan A., Cultraro C.M., Patidar R., Sindiri S., Brown A.-L. (2019). APOBEC Mutagenesis and Copy-Number Alterations Are Drivers of Proteogenomic Tumor Evolution and Heterogeneity in Metastatic Thoracic Tumors. Cell Rep..

[29] Sharpnack M.F., Ranbaduge N., Srivastava A., Cerciello F., Codreanu S.G., Liebler D.C., Mascaux C., Miles W.O., Morris R., McDermott J.E. (2018). Proteogenomic Analysis of Surgically Resected Lung Adenocarcinoma. J. Thorac. Oncol..

[30] Sankala H., Vaughan C., Wang J., Deb S., Graves P.R. (2011). Upregulation of the Mitochondrial Transport Protein, Tim50, by Mutant P53 Contributes to Cell Growth and Chemoresistance. Arch. Biochem. Biophys..

[31] Nishimura T., Nakamura H., Tan K.T., Zhuo D.-W., Fujii K., Koizumi H., Naruki S., Takagi M., Furuya N., Kato Y. (2020). A Proteogenomic Profile of Early Lung Adenocarcinomas by Protein Co-Expression Network and Genomic Alteration Analysis. Sci. Rep..

[32] Chen Y.-J., Roumeliotis T.I., Chang Y.-H., Chen C.-T., Han C.-L., Lin M.-H., Chen H.-W., Chang G.-C., Chang Y.-L., Wu C.-T. (2020). Proteogenomics of Non-Smoking Lung Cancer in East Asia Delineates Molecular Signatures of Pathogenesis and Progression. Cell.

[33] Gillette M.A., Satpathy S., Cao S., Dhanasekaran S.M., Vasaikar S.V., Krug K., Petralia F., Li Y., Liang W.-W., Reva B. (2020). Proteogenomic Characterization Reveals Therapeutic Vulnerabilities in Lung Adenocarcinoma. Cell.

[34] Soltis A.R., Bateman N.W., Liu J., Nguyen T., Franks T.J., Zhang X., Dalgard C.L., Viollet C., Somiari S., Yan C. (2022). Proteogenomic Analysis of Lung Adenocarcinoma Reveals Tumor Heterogeneity, Survival Determinants, and Therapeutically Relevant Pathways. Cell Rep. Med..

[35] Zhang Y., Fu F., Zhang Q., Li L., Liu H., Deng C., Xue Q., Zhao Y., Sun W., Han H. (2024). Evolutionary Proteogenomic Landscape from Pre-Invasive to Invasive Lung Adenocarcinoma. Cell Rep. Med..

[36] Stewart P.A., Parapatics K., Welsh E.A., Müller A.C., Cao H., Fang B., Koomen J.M., Eschrich S.A., Bennett K.L., Haura E.B. (2015). A Pilot Proteogenomic Study with Data Integration Identifies MCT1 and GLUT1 as Prognostic Markers in Lung Adenocarcinoma. PLoS ONE.

[37] Vizcaíno J.A., Deutsch E.W., Wang R., Csordas A., Reisinger F., Ríos D., Dianes J.A., Sun Z., Farrah T., Bandeira N. (2014). ProteomeXchange Provides Globally Coordinated Proteomics Data Submission and Dissemination. Nat. Biotechnol..

[38] Kikuchi T., Hassanein M., Amann J.M., Liu Q., Slebos R.J.C., Rahman S.M.J., Kaufman J.M., Zhang X., Hoeksema M.D., Harris B.K. (2012). In-Depth Proteomic Analysis of Nonsmall Cell Lung Cancer to Discover Molecular Targets and Candidate Biomarkers. Mol. Cell. Proteom..

[39] Stewart P.A., Welsh E.A., Slebos R.J.C., Fang B., Izumi V., Chambers M., Zhang G., Cen L., Pettersson F., Zhang Y. (2019). Proteogenomic Landscape of Squamous Cell Lung Cancer. Nat. Commun..

[40] Ruggles K.V., Krug K., Wang X., Clauser K.R., Wang J., Payne S.H., Fenyö D., Zhang B., Mani D.R. (2017). Methods, Tools and Current Perspectives in Proteogenomics. Mol. Cell. Proteom..

[41] Li L., Wei Y., To C., Zhu C.-Q., Tong J., Pham N.-A., Taylor P., Ignatchenko V., Ignatchenko A., Zhang W. (2014). Integrated Omic Analysis of Lung Cancer Reveals Metabolism Proteome Signatures with Prognostic Impact. Nat. Commun..

[42] Satpathy S., Krug K., Jean Beltran P.M., Savage S.R., Petralia F., Kumar-Sinha C., Dou Y., Reva B., Kane M.H., Avanessian S.C. (2021). A Proteogenomic Portrait of Lung Squamous Cell Carcinoma. Cell.

[43] Lehtiö J., Arslan T., Siavelis I., Pan Y., Socciarelli F., Berkovska O., Umer H.M., Mermelekas G., Pirmoradian M., Jönsson M. (2021). Proteogenomics of Non-Small Cell Lung Cancer Reveals Molecular Subtypes Associated with Specific Therapeutic Targets and Immune-Evasion Mechanisms. Nat. Cancer.

[44] Sica G.L., Choi I.-H., Zhu G., Tamada K., Wang S.-D., Tamura H., Chapoval A.I., Flies D.B., Bajorath J., Chen L. (2003). B7-H4, a Molecule of the B7 Family, Negatively Regulates T Cell Immunity. Immunity.

[45] Liu Q., Zhang J., Guo C., Wang M., Wang C., Yan Y., Sun L., Wang D., Zhang L., Yu H. (2024). Proteogenomic Characterization of Small Cell Lung Cancer Identifies Biological Insights and Subtype-Specific Therapeutic Strategies. Cell.

[46] Treue D., Bockmayr M., Stenzinger A., Heim D., Hester S., Klauschen F. (2019). Proteogenomic Systems Analysis Identifies Targeted Therapy Resistance Mechanisms in EGFR-mutated Lung Cancer. Int. J. Cancer.

[47] Wu J., Hao Z., Ma C., Li P., Dang L., Sun S. (2020). Comparative Proteogenomics Profiling of Non-Small and Small Lung Carcinoma Cell Lines Using Mass Spectrometry. PeerJ.

[48] Qi Y.A., Maity T.K., Cultraro C.M., Misra V., Zhang X., Ade C., Gao S., Milewski D., Nguyen K.D., Ebrahimabadi M.H. (2021). Proteogenomic Analysis Unveils the HLA Class I-Presented Immunopeptidome in Melanoma and EGFR-Mutant Lung Adenocarcinoma. Mol. Cell. Proteom..

[49] Chen X.-Y., Zhang J., Zhu J.-S. (2019). The Role of m6A RNA Methylation in Human Cancer. Mol. Cancer.

[50] Wang X., Yu Q., Yu H., Wang Y., Sun L., Yu L., Cui H., Yang H. (2022). The Prognostic Value and Multiomic Features of m6A-Related Risk Signature in Lung Adenocarcinoma. Am. J. Transl. Res..

[51] Wu L., Zhong Y., Wu D., Xu P., Ruan X., Yan J., Liu J., Li X. (2022). Immunomodulatory Factor TIM3 of Cytolytic Active Genes Affected the Survival and Prognosis of Lung Adenocarcinoma Patients by Multi-Omics Analysis. Biomedicines.

[52] Aravidis C., Panani A.D., Kosmaidou Z., Thomakos N., Rodolakis A., Antsaklis A. (2012). Detection of Numerical Abnormalities of Chromosome 9 and P16/CDKN2A Gene Alterations in Ovarian Cancer with Fish Analysis. Anticancer Res..

[53] Dagher J., Dugay F., Verhoest G., Cabillic F., Jaillard S., Henry C., Arlot-Bonnemains Y., Bensalah K., Oger E., Vigneau C. (2013). Histologic Prognostic Factors Associated with Chromosomal Imbalances in a Contemporary Series of 89 Clear Cell Renal Cell Carcinomas. Hum. Pathol..

[54] Ahn J.-M., Kim M.-S., Kim Y.-I., Jeong S.-K., Lee H.-J., Lee S.H., Paik Y.-K., Pandey A., Cho J.-Y. (2014). Proteogenomic Analysis of Human Chromosome 9-Encoded Genes from Human Samples and Lung Cancer Tissues. J. Proteome Res..

[55] Paik Y.-K., Jeong S.-K., Omenn G.S., Uhlen M., Hanash S., Cho S.Y., Lee H.-J., Na K., Choi E.-Y., Yan F. (2012). The Chromosome-Centric Human Proteome Project for Cataloging Proteins Encoded in the Genome. Nat. Biotechnol..

[56] Chang J.S., Wrensch M.R., Hansen H.M., Sison J.D., Aldrich M.C., Quesenberry C.P., Seldin M.F., Kelsey K.T., Kittles R.A., Silva G. (2008). Nucleotide Excision Repair Genes and Risk of Lung Cancer among San Francisco Bay Area Latinos and African Americans. Int. J. Cancer.

[57] Iida M., Brand T.M., Campbell D.A., Starr M.M., Luthar N., Traynor A.M., Wheeler D.L. (2013). Targeting AKT with the Allosteric AKT Inhibitor MK-2206 in Non-Small Cell Lung Cancer Cells with Acquired Resistance to Cetuximab. Cancer Biol. Ther..

[58] Moriya Y., Nohata N., Kinoshita T., Mutallip M., Okamoto T., Yoshida S., Suzuki M., Yoshino I., Seki N. (2012). Tumor Suppressive microRNA-133a Regulates Novel Molecular Networks in Lung Squamous Cell Carcinoma. J. Hum. Genet..

[59] Nishimura T., Nakamura H., Végvári Á., Marko-Varga G., Furuya N., Saji H. (2019). Current Status of Clinical Proteogenomics in Lung Cancer. Expert Rev. Proteom..

